# Real-world effectiveness and safety of blinatumomab in adults with B-cell precursor acute lymphoblastic leukaemia across 13 European countries

**DOI:** 10.1038/s41408-026-01506-x

**Published:** 2026-05-02

**Authors:** Sabina Chiaretti, Sabine Blum, Thibaut Leguay, Marie Balsat, Cyril Salek, Nicola Fracchiolla, Alexandros Spyridonidis, Anita Rijneveld, Cristina Papayannidis, Albertina Nunes, Anne Christine Wilke, Sigrid Machherndl-Spandl, Ulla Wartiovaara-Kautto, Jessica Choudhry, Ravikanth Maraboina, Gerhard Zugmaier, Noemi Mergen, Andreas Ochs, Alessandro Rambaldi

**Affiliations:** 1 Hematology Department of Translational and Precision Medicine, “Sapienza” University, Rome, Italy; 2 University Hospital and University of Lausanne, Lausanne, Switzerland; 3 University Hospital of Bordeaux, Bordeaux, France; 4 Hematology Department, Lyon-Sud Hospital, Pierre Bénite, France; 5 Institute of Hematology and Blood Transfusion, Prague, Czech Republic; 6 SC Ematologia, Fondazione IRCCS Ca’ Granda Ospedale Maggiore Policlinico, Milan, Italy; 7 University Hospital of Patras, Patra, Greece; 8 Erasmus MC Cancer Institute, Rotterdam, The Netherlands; 9 IRCCS, Azienda Ospedaliero Universitaria di Bologna, Istituto di Ematologia “L e A Seràgnoli”, Bologna, Italy; 10 Instituto Portugues de Oncologia de Lisboa Francisco Gentil, Lisbon, Portugal; 11 University Hospital Frankfurt, Frankfurt, Germany; 12 Ordensklinikum Linz Elisabethinen, Linz, Austria; 13 Department of Hematology, Helsinki University Hospital Comprehensive Cancer Center and University of Helsinki, Helsinki, Finland; 14 Amgen Inc, Thousand Oaks, CA, USA; 15 IQVIA, Bangalore, India; 16 Amgen Research Munich GmbH, Munich, Germany; 17 Amgen Ltd, Uxbridge, UK; 18 Department of Oncology and Hematology, University of Milan and Azienda Socio Sanitaria Territoriale Papa Giovanni XXIII, Bergamo, Italy

**Keywords:** Acute lymphocytic leukaemia, Immunotherapy

## Abstract

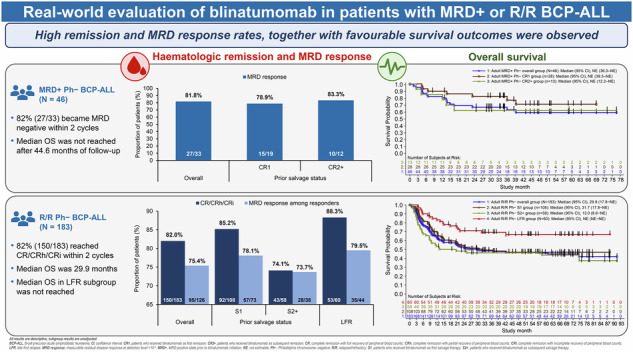

**To the Editor**,

Blinatumomab, a CD19/CD3-bispecific T-cell engager (BiTE^®^), is an established immunotherapy for treating measurable residual disease–positive (MRD+) and relapsed/refractory (R/R) Philadelphia chromosome–negative (Ph−) CD19–positive B-cell precursor acute lymphoblastic leukaemia (BCP-ALL) [1]. Following conditional marketing authorisation from the European Medicines Agency (EMA) in 2015, this post-authorisation safety study (PASS; NCT03117621) evaluated the real-world effectiveness and safety of blinatumomab in adult patients with MRD+ or R/R Ph− BCP-ALL across 78 centres in 13 European countries (Supplementary Table 1). It also included an important but under-represented subset of patients with late first relapse (LFR), defined as initial remission lasting for ≥12 months. Outcomes evaluated in the study are summarised in Supplementary Table 2.

This multicentre, retrospective, observational study reviewed 264 adult patients treated with blinatumomab between 2015 and 2022 (data collection: March 2017–March 2022) with a minimum follow-up of two years from treatment initiation. Of these, 246 adult patients with Ph− BCP-ALL were included in the effectiveness analysis: MRD+ (*n* = 46) and R/R (*n* = 183, including 60 in LFR). Median age at blinatumomab initiation was 46.0 years for both groups (LFR, 35.5 years). Detailed in Table 1, in the MRD+ group, 28 patients received blinatumomab in first remission (CR1) and 13 in subsequent remission (CR2+). No patients in CR1 and two in CR2+ had received prior allogeneic haematopoietic stem cell transplantation (allo-HSCT). In the R/R group, 108 patients received blinatumomab as first salvage (S1) and 58 as later salvage therapy (S2+). Additionally, 38 patients in the R/R group (including 17 in LFR) received prior allo-HSCT (Supplementary Table 3). Patients completed a median of 2 (range, 1–9) blinatumomab cycles (Supplementary Table 4).

**Table 1:** Baseline disease and treatment characteristics by disease subgroups.

Characteristics	Adult MRD+ Ph− BCP-ALL			Adult R/R Ph− BCP-ALL			
	Overall (*N* = 46)	Prior remission status among overall^a^		Overall (*N* = 183)	Prior salvage status among overall^a^		LFR subset (*N* = 60)
		CR1 (*N* = 28)	CR2+ (*N* = 13)		S1 (*N* = 108)	S2+ (*N* = 58)	
**Age at ALL diagnosis (years), median (min–max)**	42.5 (17.0–70.0)	42.5 (19.0–69.0)	43.0 (17.0–68.0)	44.0 (0.2–79.0)	45.5 (13.0–79.0)	42.0 (0.2–76.0)	32.0 (15.0–79.0)
**Disease Characteristics**							
**BCP-ALL subtype, n (%)**							
Pro–BCP-ALL	12 (26.1)	9 (32.1)	2 (15.4)	36 (19.7)	21 (19.4)	13 (22.4)	7 (11.7)
Pre–BCP-ALL	10 (21.7)	5 (17.9)	3 (23.1)	47 (25.7)	29 (26.9)	15 (25.9)	17 (28.3)
C-ALL	15 (32.6)	9 (32.1)	4 (30.8)	55 (30.1)	32 (29.6)	17 (29.3)	19 (31.7)
BCP-ALL with recurrent genetic abnormality	9 (19.6)	5 (17.9)	4 (30.8)	33 (18.0)	21 (19.4)	8 (13.8)	10 (16.7)
Missing	0 (0.0)	0 (0.0)	0 (0.0)	12 (6.6)	5 (4.6)	5 (8.6)	7 (11.7)
**WBC, n (%)**							
<30,000/µL	30 (65.2)	18 (64.3)	9 (69.2)	108 (59.0)	63 (58.3)	37 (63.8)	33 (55.0)
≥30,000/µL	12 (26.1)	9 (32.1)	2 (15.4)	53 (29.0)	33 (30.6)	12 (20.7)	18 (30.0)
Unknown	4 (8.7)	1 (3.6)	2 (15.4)	20 (10.9)	10 (9.3)	9 (15.5)	8 (13.3)
Missing	0 (0.0)	0 (0.0)	0 (0.0)	2 (1.1)	2 (1.9)	0 (0.0)	1 (1.7)
**Extramedullary disease, n (%)**	12 (26.1)	5 (17.9)	6 (46.2)	20 (10.9)	11 (10.2)	7 (12.1)	8 (13.3)
CNS	5 (10.9)	1 (3.6)	4 (30.8)	9 (4.9)	5 (4.6)	3 (5.2)	4 (6.7)
Testis	0 (0.0)	0 (0.0)	0 (0.0)	1 (0.5)	1 (0.9)	0 (0.0)	1 (1.7)
Other	7 (15.2)	4 (14.3)	2 (15.4)	10 (5.5)	5 (4.6)	4 (6.9)	3 (5.0)
Missing	0 (0.0)	0 (0.0)	0 (0.0)	1 (0.5)	0 (0.0)	0 (0.0)	0 (0.0)
**Disease status at time of blinatumomab initiation, n (%)**							
Primary refractory	0 (0.0)	0 (0.0)	0 (0.0)	23 (12.6)	23 (21.3)	0 (0.0)	0 (0.0)
Refractory to salvage therapy	0 (0.0)	0 (0.0)	0 (0.0)	33 (18.0)	0 (0.0)	33 (56.9)	NA
Missing	0 (0.0)	0 (0.0)	0 (0.0)	4 (2.2)	3 (2.8)	1 (1.7)	NA
Late first relapse (duration of prior remission >12 months)	0 (0.0)	0 (0.0)	0 (0.0)	60 (32.8)	47 (43.5)	5 (8.6)	60 (100.0)
Missing	0 (0.0)	0 (0.0)	0 (0.0)	3 (1.6)	3 (2.8)	0 (0.0)	0 (0.0)
Untreated second or greater relapse	0 (0.0)	0 (0.0)	0 (0.0)	16 (8.7)	0 (0.0)	12 (20.7)	0 (0.0)
Missing	0 (0.0)	0 (0.0)	0 (0.0)	3 (1.6)	3 (2.8)	0 (0.0)	0 (0.0)
Morphological CR MRD+	46 (100.0)	28 (100.0)	13 (100.0)	0 (0.0)	0 (0.0)	0 (0.0)	0 (0.0)
Missing	0 (0.0)	0 (0.0)	0 (0.0)	10 (5.5)	8 (7.4)	2 (3.4)	2 (3.3)
Untreated ALL in CNS	3 (6.5)	3 (10.7)	0 (0.0)	6 (3.3)	4 (3.7)	2 (3.4)	2 (3.3)
Missing	8 (17.4)	6 (21.4)	2 (15.4)	44 (24.0)	25 (23.1)	12 (20.7)	15 (25.0)
**Prior Treatment History**							
**HSCT before blinatumomab, n (%)**	5 (10.9)	0 (0.0)	2 (15.4)	39 (21.3)	17 (15.7)	12 (20.7)	17 (28.3)
Autologous	1 (2.2)	0 (0.0)	1 (7.7)	1 (0.5)	1 (0.9)	0 (0.0)	0 (0.0)
Allogeneic	4 (8.7)	0 (0.0)	1 (7.7)	38 (20.8)	16 (14.8)	12 (20.7)	17 (28.3)
**Time from previous HSCT to start of blinatumomab (months), median (min–max)**	10.7 (4.2–53.9)	NA	29.9 (5.8–53.9)	10.9 (2.5–137.7)	10.9 (3.8–77.1)	6.2 (2.4–137.7)	17.3 (8.5–77.1)
**Prior lines of anti-cancer therapy**^**b**^ **for ALL, n (%)**							
First line	28 (60.9)	28 (100.0)	0 (0.0)	108 (59.0)	108 (100.0)	0 (0.0)	47 (78.3)
Second line	9 (19.6)	0 (0.0)	9 (69.2)	44 (24.0)	0 (0.0)	44 (75.9)	5 (8.3)
Third line	4 (8.7)	0 (0.0)	4 (30.8)	13 (7.1)	0 (0.0)	13 (22.4)	0 (0.0)
Fourth line	0 (0.0)	0 (0.0)	0 (0.0)	1 (0.5)	0 (0.0)	1 (1.7)	0 (0.0)
Conditioning regimen for HSCT	2 (4.3)	0 (0.0)	0 (0.0)	8 (4.4)	0 (0.0)	0 (0.0)	5 (8.3)
Other	3 (6.5)	0 (0.0)	0 (0.0)	9 (4.9)	0 (0.0)	0 (0.0)	3 (5.0)
**Treatment Characteristics**							
**Number of cycles started, median (min–max)**	2 (1–6)	2 (1–6)	2 (1–3)	2 (1–9)	2 (1–9)	2 (1–5)	2 (1–9)
**Duration of treatment (days), median (min–max)**	57 (16.0–172.0)	57.5 (16.0–172.0)	57 (29.0–88.0)	57 (6.0–215.0)	58 (6.0–215.0)	56 (11.0–149.0)	58.0 (13.0–215.0)
**Duration of treatment who had HSCT prior to blinatumomab (days), median (min–max)**	56.5 (29.0–57.0)	NA	57 (57.0–57.0)	67 (13.0–215.0)	85.5 (13.0–215.0)	58.5 (12.0–145.0)	61.0 (13.0–215.0)
**Duration of treatment who did not have HSCT prior to blinatumomab (days), median (min–max)**	57.5 (16.0–172.0)	57.5 (16.0–172.0)	57.5 (29.0–88.0)	56 (6.0–156.0)	57.5 (6.0–156.0)	47.5 (11.0–149.0)	58.0 (25.0–149.0)

*ALL* acute lymphoblastic leukaemia, *BCP-ALL* B-cell precursor acute lymphoblastic leukaemia, *C-ALL* common acute lymphoblastic leukaemia, *CNS* central nervous system, *CR1* patients who received blinatumomab as the first remission, *CR2*+ patients who received blinatumomab as subsequent remission, *CR* complete remission, *HSCT* haematopoietic stem cell transplant, *LFR* late first relapse, *max* maximum, *min* minimum, *MRD* measurable residual disease, *NA* not applicable, *Ph* Philadelphia chromosome, *R/R* relapsed/refractory, *S1* patients who received blinatumomab as first salvage therapy, *S2*+ patients who received blinatumomab as subsequent salvage therapy, *WBC* white blood cell.

^a^Does not include patients under ‘conditioning regimen for HSCT/others’ (5 in MRD+ and 17 in R/R groups).

^b^In patients with MRD+ Ph− BCP-ALL, prior lines of therapy were earlier treatments leading to remission and in patients with R/R Ph− BCP-ALL, prior lines of therapy were salvage therapies.

In the MRD+ group (*n* = 46), 27 (81.8%) of 33 evaluable patients achieved an MRD response, with similar rates across the CR1 and CR2+ subgroups (Fig. 1A). Median disease-free survival (DFS) was 31.2 months (95% confidence interval [CI], 5.9–not estimable [NE]) and was NE for other subgroups (Supplementary Table 5). After a median follow-up of 44.6 months, overall survival (OS) was not reached (Fig. 1B). The 24-month OS was 67% (95% CI, 51–79) overall and 54% (95% CI, 24–77) with censoring at allo-HSCT; it was more favourable for patients in CR1 than in CR2+ (82% [95% CI, 62–92] vs 62% [95% CI, 31–82]; Supplementary Table 6).

**Fig. 1:**
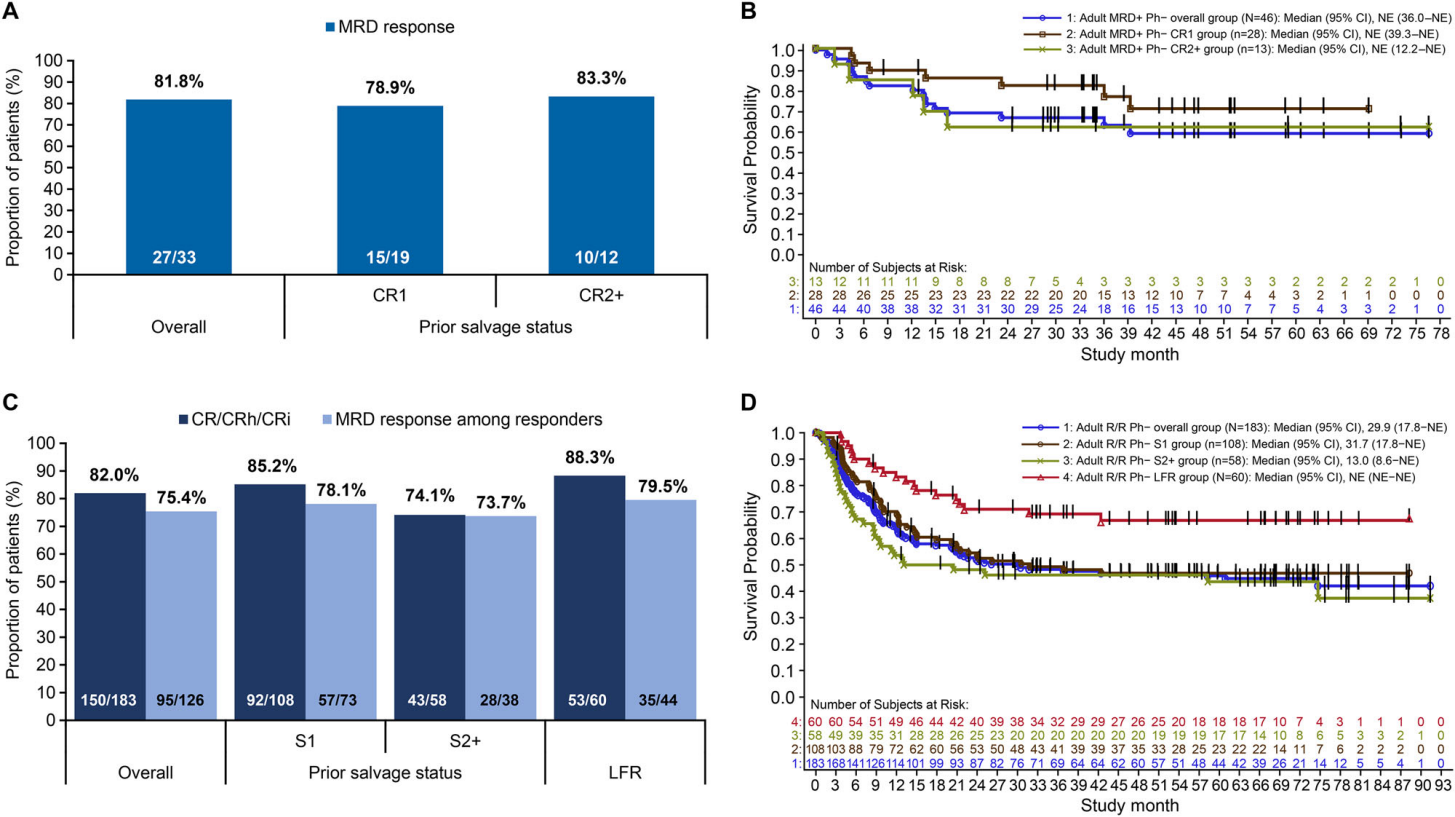
Response and survival outcomes in patients with MRD+ Ph– BCP-ALL or R/R Ph– BCP-ALL. **A** MRD response among adult patients with evaluable MRD in the MRD+ Ph– BCP-ALL group within the first two cycles of blinatumomab treatment. **B** Kaplan–Meier analysis of overall survival in patients with MRD+ Ph– BCP-ALL following blinatumomab treatment. **C** Response outcomes in the R/R Ph– BCP-ALL group and subgroups within the first two cycles of blinatumomab treatment. **D** Kaplan–Meier analysis of overall survival in patients with R/R Ph– BCP-ALL following blinatumomab treatment. BCP-ALL B-cell precursor acute lymphoblastic leukaemia, CI confidence interval, CR1 patients who received blinatumomab as first remission, CR2+ patients who received blinatumomab as subsequent remission, CR complete remission with full recovery of peripheral blood counts, CRh complete remission with partial recovery of peripheral blood counts, CRi complete remission with incomplete recovery of peripheral blood counts, LFR late first relapse, MRD measurable residual disease, NE not estimable, Ph Philadelphia chromosome, R/R relapsed/refractory, S1 patients who received blinatumomab as first salvage therapy, S2+ patients who received blinatumomab as subsequent salvage therapy.

In the R/R group (*n* = 183), 150 patients (82.0%) achieved complete remission (CR), CR with partial haematologic recovery (CRh), or CR with incomplete haematologic recovery (CRi), including 124 (82.7%) achieving CR. Among 126 MRD-evaluable patients, 95 (75.4%) achieved an MRD response. Both CR/CRh/CRi and MRD response rates were higher in patients in S1 than in S2+ (Fig. 1C). As shown in Supplementary Fig. 1, a trend towards better CR/CRh/CRi response was observed in patients with prior allo-HSCT (89.5%) than without (80.0%); however, a lower proportion of these achieved CR, while MRD responses were similar irrespective of prior allo-HSCT status. Outcomes in the LFR subset were better than those in the overall group, as 53 patients (88.3%) achieved CR/CRh/CRi and 35 (79.5%) of 44 MRD-evaluable patients achieved an MRD response.

In the R/R group, median relapse-free survival (RFS) was 15.9 months (95% CI, 8.7–41.3) and OS was 29.9 months (95% CI, 17.8–NE); both endpoints were not reached in the LFR subset (Supplementary Tables 7 and 8). MRD responders had longer median RFS than non-responders (20.5 months vs 6.5 months). The overall 24-month RFS was 43% (95% CI, 35–51) and was similar across subgroups. The 24-month OS was 52% (95% CI, 44–59) overall and 71% (95% CI, 58–81) among LFR patients, with lower estimates when censored at allo-HSCT (Supplementary Table 8). Earlier administration of blinatumomab was associated with longer RFS (S1 vs S2+, 16.0 months vs 8.7 months) and OS (S1 vs S2+, 31.7 months vs 13.0 months; Fig. 1D). Patients without prior allo-HSCT had longer RFS than those with prior allo-HSCT (18.5 months vs 11.3 months); however, these results might be confounded by differences in S1 and S2+ distribution. In contrast, prior allo-HSCT was associated with a substantially prolonged OS (74.7 months vs 22.6 months).

Following blinatumomab, a substantial proportion of patients in both disease groups proceeded to allo-HSCT; 33/46 (71.7%) MRD+ patients and 92/150 (61.3%, including 33/53 [62.2%] in LFR) R/R patients in CR/CRh/CRi. Most patients did not require additional anti-cancer therapy prior to allo-HSCT (MRD+, 28/33 [84.8%]; R/R, 64/92 [69.6%]; LFR, 27/33 [81.8%]; Supplementary Tables 9 and 10).

The full safety analysis set (*N* = 264) included 35 additional patients with Ph+ BCP-ALL and 21 with other ALL diagnoses. Treatment-related treatment-emergent adverse events (TR-TEAEs) occurred in 182 patients (68.9%), with comparable incidence across disease groups (Supplementary Table 11). Grade ≥3 TR-TEAEs occurred in 91 patients (34.5%), serious TR-TEAEs in 77 patients (29.2%), and fatal TR-TEAEs in two patients (respiratory failure secondary to CRS and neurotoxicity). The most common ( ≥ 10%) TR-TEAEs were pyrexia (27.7%), CRS (14.4%), and headache (11.4%). Among TR-TEAEs of interest, neurologic (28.0%; grade ≥3, 8.0%), CRS (14.4%; grade ≥3, 3.8%), and opportunistic infections (1.5%; grade ≥3, 0.4%) were reported in the entire study. Over half of the patients experienced no dose interruptions; among those who did, it was primarily due to AEs (32.6%). Blinatumomab-related treatment discontinuation occurred in 13 patients (4.9%). Blinatumomab interruption and discontinuation were higher in elderly patients (Supplementary Table 12), likely due to higher prevalence of comorbidities and generally increased caution in treating this patient population. Additionally, 31 medication errors were reported for 26 patients (9.8%), most commonly administration-related, primarily due to operator use error and pump malfunction (Supplementary Table 13).

Overall, this is the largest reported real-world study of blinatumomab-treated patients to date. High haematologic remission and MRD response rates were observed within two cycles. Notably, in both disease groups, higher response rates and improved survival outcomes were associated with earlier blinatumomab use, consistent with clinical and real-world data [2–4]. Response in the MRD+ group was similar to previous reports [3, 5–7] and was associated with improved RFS, reinforcing the established prognostic importance of MRD response [8]. In the R/R group, response rates exceeded those reported in early single-arm clinical trials [9, 10] and subsequent real-world studies [3, 6], likely reflecting differences in patient-risk and pre-treatment profiles, earlier use of blinatumomab as salvage therapy, and increasing physician experience in clinical practice. MRD response in this group was independent of prior allo-HSCT, indicating that transplant status does not limit MRD response potential.

Survival outcomes further supported the response findings. Median RFS was similar to published reports [3, 7], while median OS exceeded that reported in other studies [3, 7, 9], potentially reflecting a lower proportion of pre-treated patients, higher initial response to blinatumomab, and limited capture of deaths in community practice. A short RFS among MRD non-responders reinforces the prognostic importance of achieving an MRD response [8]. Patients in the LFR subset achieved higher response rates and better survival outcomes than the overall R/R population, consistent with previous findings, suggesting favourable disease biology and preserved immune function in late relapse [11, 12]. In the R/R group, a prolonged OS in patients with prior allo-HSCT may reflect enhanced blinatumomab activity facilitated by donor-derived T-cells [13]. However, a shorter RFS in these patients did not translate to meaningful difference in the 24-month OS between allo-HSCT recipients and non-recipients. This could be due to donor-derived immune reactivation by blinatumomab as well as differences in more intensive post-relapse management in this likely relatively fitter subgroup.

Following blinatumomab, a higher proportion of patients in both the disease groups proceeded to allo-HSCT than in NEUF, with similar reported mortality [3]. Most patients underwent allo-HSCT without any prior anti-cancer therapy, highlighting the role of blinatumomab as an effective bridge to transplant as well as an effective therapy irrespective of prior transplant status.

The safety profile of blinatumomab was consistent with clinical trials [1, 2, 9, 14] and real-world studies [3, 6, 7], with no new safety signals identified. Among TR-TEAEs of interest, neurologic events were lower than clinical trial reports and closely aligned with the real-world estimate while CRS events were comparable with clinical data and lower than real-world data [1, 6]. Similar to previous reports [5, 9, 14], most TR-TEAEs resolved completely with supportive care and management. Blinatumomab associated discontinuation was also lower than in previous reports [6, 9]. These findings suggest an increased experience with blinatumomab administration and TR-TEAE management. Administration-related medication errors associated with infusion pump use highlight the potential of exploring alternative approaches, such as subcutaneous formulations [15].

The large study size and inclusion of clinically relevant subgroups strengthen the external validity and generalisability of these findings, although limitations inherent to retrospective real-world analyses include selection bias, variability in MRD assessment methods, and potential incomplete capture of outcomes occurring outside treating hospitals. In conclusion, high remission and MRD response rates, together with favourable survival outcomes, were observed in patients with MRD+ or R/R Ph− BCP-ALL treated in routine clinical practice, including patients who were earlier in their line of treatment for BCP-ALL, reaffirming the role of blinatumomab as an effective therapy across both disease settings.

## Supplementary information

Supplemental information

AJ Checklist

## Data Availability

Qualified researchers may request data from Amgen clinical studies. Further details are available at Amgen’s website on https://wwwext.amgen.com/science/clinical-trials/clinical-data-transparency-practices/clinical-trial-data-sharing-request.
