# Supplementary material for: Real-world effectiveness and safety of blinatumomab in adults with B-cell precursor acute lymphoblastic leukaemia across 13 European countries
Source: Blood Cancer J. 2026 May 2;16(1):68. doi: 10.1038/s41408-026-01506-x (PMC13135500; doi:10.1038/s41408-026-01506-x)
Supplement: Supplementary file 1 — Supplemental information [file 41408_2026_1506_MOESM1_ESM.pdf]

SUPPLEMENTARY INFORMATION

Real-world effectiveness and safety of blinatumomab in adults with B-cell precursor acute lymphoblastic leukaemia across 13 European countries

Sabina Chiaretti[1], Sabine Blum[2], Thibaut Leguay[3], Marie Balsat[4], Cyril Salek[5], Nicola Fracchiolla[6], Alexandros Spyridonidis[7], Anita Rijneveld[8], Cristina Papayannidis[9], Albertina Nunes[10], Anne Christine Wilke[11], Sigrid Machherndl-Spandl[12], Ulla Wartiovaara-Kautto[13] Jessica Choudhry[14], Ravikanth Maraboina[15], Gerhard Zugmaier[16], Noemi Mergen[16], Andreas Ochs[17], Alessandro Rambaldi[18]

[1]Hematology Department of Translational and Precision Medicine, “Sapienza” University, Rome, Italy, [2]University Hospital and University of Lausanne, Lausanne, Switzerland, [3]University Hospital of Bordeaux, Bordeaux, France, [4]Hematology Department, Lyon-Sud Hospital, Pierre Bénite, France, [5]Institute of Hematology and Blood Transfusion, Prague, Czech Republic [6]SC Ematologia, Fondazione IRCCS Ca’ Granda Ospedale Maggiore Policlinico, Milan, Italy [7]University Hospital of Patras, Patra, Greece, [8]Erasmus MC Cancer Institute, Rotterdam, The Netherlands, [9]IRCCS, Azienda Ospedaliero Universitaria di Bologna, Istituto di Ematologia “L e A Seràgnoli”, Bologna, Italy, [10]Instituto Portugues de Oncologia de Lisboa Francisco Gentil, Lisbon, Portugal, [11]University Hospital Frankfurt, Frankfurt, Germany, [12]Ordensklinikum Linz Elisabethinen, Linz, Austria, [13]Department of Hematology, Helsinki University Hospital Comprehensive Cancer Center and University of Helsinki, Helsinki, Finland, [14]Amgen Inc, Thousand Oaks, US, [15]IQVIA, Bangalore, India, [16]Amgen Research Munich GmbH, Munich, Germany, [17]Amgen Ltd, Uxbridge, UK, [18]Department of Oncology and Hematology, University of Milan and Azienda Socio Sanitaria Territoriale Papa Giovanni XXIII, Bergamo, Italy

TABLE OF CONTENTS

Supplementary Figure 1. Response outcomes for patients with R/R Ph– BCP-ALL, overall and by sub-groups within the first two cycles of blinatumomab treatment. 3

Supplementary Table 1. Distribution of participating study centres 4

Supplementary Table 2. Effectiveness and safety outcomes in the study 5

Supplementary Table 3. Disease and treatment characteristics in patients with R/R Ph− BCP-ALL with prior allo-HSCT 6

Supplementary Table 4. Treatment characteristics 8

Supplementary Table 5. Disease-free survival outcomes in patients with MRD+ Ph− BCP-ALL by subgroups 9

Supplementary Table 6. Overall survival outcomes in patients with MRD+ Ph− BCP-ALL by subgroups 10

Supplementary Table 7. Relapse-free survival outcomes among CR/CRh/CRi responders in patients with R/R Ph− BCP-ALL by subgroups...11

Supplementary Table 8. Overall survival outcomes in patients with R/R Ph− BCP-ALL by subgroups 12

Supplementary Table 9. HSCT following blinatumomab treatment in adult patients with MRD+ Ph− BCP-ALL 13

Supplementary Table 10. HSCT following blinatumomab treatment in adult patients with R/R Ph− B-ALL 15

Supplementary Table 11. Safety outcomes...17

Supplementary Table 12. Incidence of adverse events by age 19

Supplementary Table 13. Description of medication errors in the study 21

Supplementary Figure 1. Response outcomes for patients with R/R Ph– BCP-ALL, overall and by sub-groups within the first two cycles of blinatumomab treatment.

Allo-HSCT, allogeneic haematopoietic stem cell transplantation; CR, complete remission with full recovery of peripheral blood counts; CRh, complete remission with partial recovery of peripheral blood counts; CRi, complete remission with incomplete recovery of peripheral blood counts; LFR, late first relapse; MRD, measurable residual disease; n, number of patients achieving the specified outcome; N, total number of evaluable patients in each subgroup; S1, patients who received blinatumomab as first salvage therapy; S2+, patients who received blinatumomab as subsequent salvage therapy.

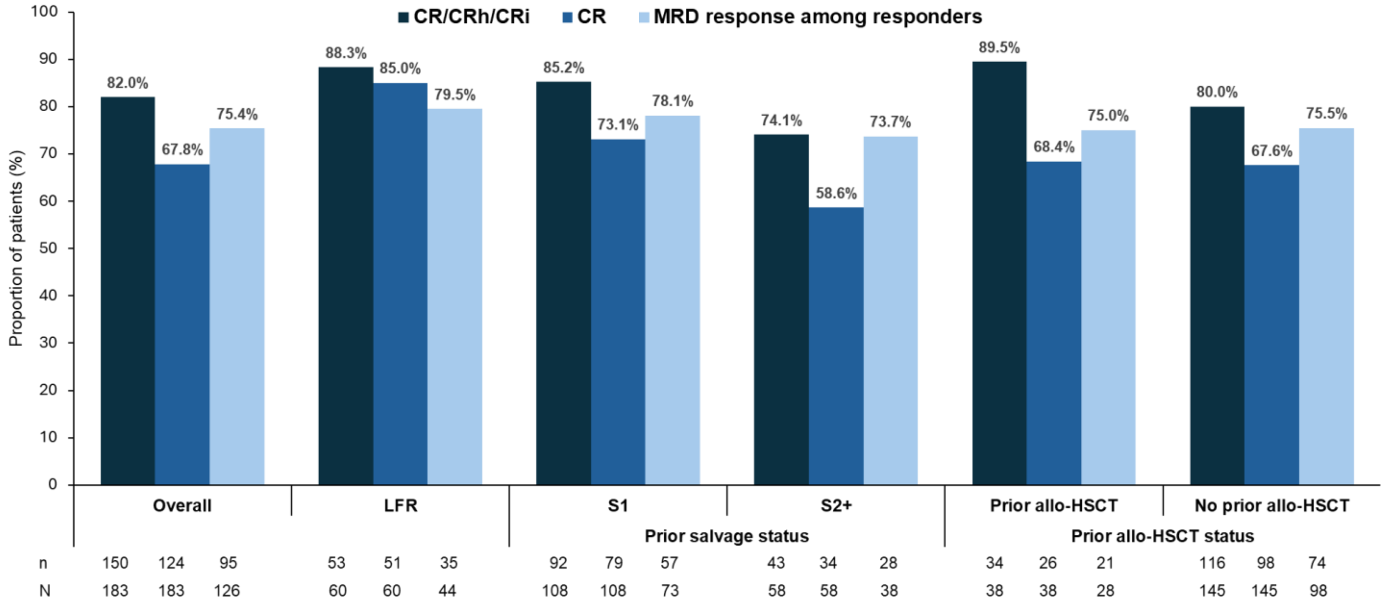

Supplementary Table 1. Distribution of participating study centres

The study was approved by local institutional review boards/independent ethics committees and was conducted in accordance with the Declaration of Helsinki and Good Clinical Practice guidelines issued by the International Council for Harmonisation. In countries where patient informed consent was required to access medical records, medical records of patients who did not provide informed consent were excluded.

Study country	Number of centres
Austria	3
Czech Republic	3
Finland	1
France	15
Germany	5
Greece	4
Italy	17
Poland	6
Portugal	5
Sweden	1
Switzerland	9
The Netherlands	3
United Kingdom	6

Supplementary Table 2. Effectiveness and safety outcomes in the study

**Outcomes**	**Definition**
**Complete remission (CR)**	Bone marrow blasts <5%, platelets >100,000 cells/μL, and absolute neutrophil count >1,000 cells/μL
**Complete remission with partial haematologic recovery (CRh)**	Bone marrow blasts ≤5%, platelets >50,000 cells/μL, and absolute neutrophil count >500 cells/μL
**Complete remission with incomplete haematologic recovery (CRi)**	Bone marrow blasts ≤5% and incomplete recovery of peripheral blood counts
**Disease-free survival (DFS)**	Time from blinatumomab initiation until relapse or death
**Measurable Residual Disease (MRD) response**	Evaluated at a level of $<10^{-4}$, assessed by polymerase chain reaction, flow cytometry, or next-generation sequencing
**Overall Survival (OS)**	Time from blinatumomab initiation until death
**Relapse**	Bone marrow blasts >5% or blasts in peripheral blood after documented CR/CRh/CRi
**Relapse-free survival (RFS)**	Time from CR/CRh/CRi until relapse or death
**Treatment-emergent adverse events (TEAEs)**	Adverse events occurring between blinatumomab initiation and 30 days after the last infusion
**Treatment-related TEAEs (TR-TEAEs)**	TEAEs considered by the treating physician to be related to blinatumomab

Supplementary Table 3. Disease and treatment characteristics in patients with R/R Ph− BCP-ALL with prior allo-HSCT

	Adult R/R Ph− BCP-ALL (N=38)	Adult R/R Ph− BCP-ALL LFR (N=17)
Age at ALL diagnosis (years), median (min–max)	33.5 (16.0–62.0)	31.0 (18.0–55.0)
**Disease Characteristics**		
**BCP-ALL subtype, n (%)**		
Pro-BCP-ALL	8 (21.1)	4 (23.5)
Pre-BCP-ALL	11 (28.9)	5 (29.4)
C-ALL	7 (18.4)	3 (17.6)
BCP-ALL with recurrent genetic abnormality	8 (21.1)	3 (17.6)
Missing	4 (10.5)	2 (11.8)
**WBC, n (%)**		
<30,000/μL	19 (50.0)	8 (47.1)
≥30,000/μL	12 (31.6)	8 (47.1)
Unknown	7 (18.4)	1 (5.9)
**Extramedullary disease, n (%)**	4 (10.5)	1 (5.9)
CNS	3 (7.9)	1 (5.9)
Testis	0 (0.0)	0 (0.0)
Other	1 (2.6)	0 (0.0)
**Disease status at time of blinatumomab initiation, n (%)**		
Primary refractory	0 (0.0)	0 (0.0)
Refractory to salvage therapy	3 (7.9)	0 (0.0)
Missing	0 (0.0)	0 (0.0)

Late first relapse (duration of prior remission >12 months)	17 (44.7)	17 (100.0)
Untreated second or greater relapse	11 (28.9)	0 (0.0)
Missing	0 (0.0)	0 (0.0)
Morphological CR MRD+	0 (0.0)	0 (0.0)
Missing	2 (5.3)	1 (5.9)
Untreated ALL in CNS	0 (0.0)	0 (0.0)
Missing	6 (15.8)	3 (17.6)
**Prior Treatment History**		
**Previous HSCT, n (%)**	38 (100.0)	17 (100.0)
Autologous	0 (0.0)	0 (0.0)
Allogeneic	38 (100.0)	17 (100.0)
**Time from previous HSCT to start of blinatumomab (months), median (min–max)**	12.1 (2.4–137.7)	17.3 (8.5–77.1)
**Prior lines of anti-cancer therapy for ALL, n (%)**		
Frontline chemotherapy	16 (42.1)	11 (64.7)
First salvage chemotherapy	8 (21.1)	0 (0.0)
Second salvage chemotherapy	3 (7.9)	0 (0.0)
Third or later salvage chemotherapy	1 (2.6)	0 (0.0)
Conditioning regimen for HSCT	8 (21.1)	5 (29.4)
Other	2 (5.3)	1 (5.9)
**Treatment Characteristics**		
Number of cycles started, median (min–max)	2.5 (1–9)	2 (1–9)
Duration of treatment (days), median (min–max)	67 (12.0–215.0)	61 (13.0–215.0)

ALL, acute lymphoblastic leukaemia; allo-HSCT, allogeneic haematopoietic stem cell transplant; BCP-ALL, B-cell precursor acute lymphoblastic leukaemia; C-ALL, common acute lymphoblastic leukaemia; CNS, central nervous system; CR, complete remission; HSCT, haematopoietic stem cell transplant; max, maximum; min, minimum; NA, not applicable; Ph, Philadelphia chromosome; WBC, white blood cell.

Supplementary Table 4. Treatment characteristics

Characteristics	Entire Study Cohort (N=264)	Adult R/R Ph− BCP-ALL (N=183)		Adult MRD+ Ph− BCP-ALL (N=46)
		Overall (N=183)	LFR (N=60)	
Number of cycles started, median (min–max)	2.0 (1.0–9.0)	2.0 (1.0–9.0)	2.0 (1.0–9.0)	2.0 (1.0–6.0)
Duration of treatment (days), median (min–max)	57.0 (6.0–215.0)	57.0 (6.0–215.0)	58.0 (13.0–215.0)	57.0 (16.0–172.0)
Duration of treatment in patients with HSCT prior to blinatumomab (days), median (min–max)	59.0 (12.0–215.0)	67.0 (13.0–215.0)	61.0 (13.0–215.0)	56.5 (29.0–57.0)
Duration of treatment in patients who did not have HSCT prior to blinatumomab (days), median (min–max)	56.5 (6.0–172.0)	56.0 (6.0–156.0)	58.0 (25.0–149.0)	57.5 (16.0–172.0)
**Dose interruptions, n (%)**				
None	150 (56.8)	95 (51.9)	37 (61.7)	31 (67.4)
Adverse event	86 (32.6)	68 (37.2)	17 (28.3)	10 (21.7)
Dose administration error	2 (0.8)	1 (0.5)	0 (0.0)	1 (2.2)
Patient request	2 (0.8)	2 (1.1)	0 (0.0)	0 (0.0)
Device complaint	2 (0.8)	1 (0.5)	0 (0.0)	0 (0.0)
Infusion bag emptied prematurely	1 (0.4)	1 (0.5)	0 (0.0)	0 (0.0)
Other	37 (14.0)	27 (14.8)	10 (16.7)	6 (13.0)

BCP-ALL, B-cell precursor acute lymphoblastic leukaemia; HSCT, haematopoietic stem cell transplant; LFR, late first relapse; max, maximum; min, minimum; MRD, measurable residual disease; Ph, Philadelphia chromosome; R/R, relapsed/refractory.

Supplementary Table 5. Disease-free survival outcomes in patients with MRD+ Ph− BCP-ALL by subgroups

	Adult MRD+ Ph− BCP-ALL (N=46)			
	**Death, n (%)**	**Relapse, n (%)**	**Time to Death or Relapse (Months), Median (95% CI)**	**KM Estimate at 24 Months, % (95% CI)**
**All patients (N=46)**	5 (10.9)	18 (39.1)	31.2 (5.9–NE)	54 (39–67)
**CR1 (n=28)**	2 (7.1)	10 (35.7)	NE (11.4–NE)	64 (44–79)
**CR2+ (n=13)**	2 (15.4)	4 (30.8)	NE (2.8–NE)	54 (25–76)
**Prior allo-HSCT (n=4)**	1 (25.0)	3 (75.0)	2.7 (1.4–NE)	0 (NE–NE)
**No prior allo-HSCT (n=42)**	4 (9.5)	15 (35.7)	NE (11.4–NE)	60 (43–73)

allo-HSCT, allogeneic haematopoietic stem cell transplant; BCP-ALL, B-cell precursor acute lymphoblastic leukaemia; CI, confidence interval; CR1, patients who received blinatumomab as the first remission; CR2, patients who received blinatumomab as subsequent remissions; KM, Kaplan–Meier; MRD, measurable residual disease; NE, not estimable; Ph, Philadelphia chromosome.

Supplementary Table 6. Overall survival outcomes in patients with MRD+ Ph− BCP-ALL by subgroups

	Adult MRD+ Ph− BCP-ALL (N=46)					
	**Overall survival**			**Overall survival (Censored at the time of allo-HSCT)**		
	**Death, n (%)**	**Time to death (months), median (95% CI)**	**KM estimate at 24 months, % (95% CI)**	**Death, n (%)**	**Time to death (months), median (95% CI)**	**KM estimate at 24 months, % (95% CI)**
**All patients (N=46)**	17 (37.0)	NE (36.0–NE)	67 (51–79)	8 (17.4)	NE (13.7–NE)	54 (24–77)
**CR1 (n=28)**	7 (25.0)	NE (39.3–NE)	82 (62–92)	2 (7.1)	NE (23.2–NE)	73 (24–93)
**CR2+ (n=13)**	5 (38.5)	NE (12.2–NE)	62 (31–82)	2 (15.4)	NE (4.2–NE)	76 (31–94)
**Prior allo-HSCT (n=4)**	4 (100.0)	5.4 (1.4–NE)	0 (NE–NE)	3 (75.0)	4.5 (1.4–NE)	0 (NE–NE)
**No prior allo-HSCT (n=42)**	13 (31.0)	NE (39.3–NE)	73 (57–84)	5 (11.9)	NE (13.7–NE)	62 (26–84)

allo-HSCT, allogeneic haematopoietic stem cell transplant; BCP-ALL, B-cell precursor acute lymphoblastic leukaemia; CI, confidence interval; CR1, patients who received blinatumomab as the first remission; CR2+, patients who received blinatumomab as subsequent remissions; KM, Kaplan–Meier; MRD, measurable residual disease; NE, not estimable; Ph, Philadelphia chromosome.

Supplementary Table 7. Relapse-free survival outcomes among CR/CRh/CRi responders in patients with R/R Ph− BCP-ALL by subgroups

	Adult R/R Ph− BCP-ALL (N=183)				
	**N**	**Death, n (%)**	**Relapse, n (%)**	**Time to death or relapse (months), median (95% CI)**	**KM estimate at 24 months, % (95% CI)**
**Overall**	150	29 (19.3)	61 (40.7)	15.9 (8.7–41.3)	43 (35–51)
MRD response	102	18 (17.6)	38 (37.3)	20.5 (10.5–NE)	50 (39–59)
No MRD response	28	5 (17.9)	15 (53.6)	6.5 (1.8–22.5)	29 (14–47)
**LFR**	53	5 (9.4)	19 (35.8)	NE (11.3–NE)	58 (43–70)
**S1**	92	16 (17.4)	38 (41.3)	16.0 (8.9–45.4)	42 (32–52)
**S2+**	41	5 (12.2)	16 (39.0)	8.7 (6.5–73.3)	42 (27–56)
**Prior allo-HSCT**	34	4 (11.8)	18 (52.9)	11.3 (6.0–73.3)	41 (25–57)
**No prior allo-HSCT**	116	25 (21.6)	43 (37.1)	18.5 (8.9–41.4)	44 (34–53)

allo-HSCT, allogeneic haematopoietic stem cell transplant; BCP-ALL, B-cell precursor acute lymphoblastic leukaemia; CI, confidence interval; CR, complete remission with full recovery of peripheral blood counts; CRh, complete remission with partial recovery of peripheral blood counts; CRi, complete remission with incomplete recovery of peripheral blood counts; KM, Kaplan–Meier; LFR, late first relapse; MRD, measurable residual disease; NE, not estimable; Ph, Philadelphia chromosome; R/R, relapsed/refractory; S1, patients who received blinatumomab as first salvage therapy; S2+, patients who received blinatumomab as subsequent salvage therapy.

Supplementary Table 8. Overall survival outcomes in patients with R/R Ph− BCP-ALL by subgroups

	**Adult R/R Ph− BCP-ALL (N=183)**							
	**Overall survival**				**Overall Survival (Censored at the Time of HSCT)**			
	**N**	**Death, n (%)**	**Time to death (months), median (95% CI)**	**KM estimate at 24 months, % (95% CI)**	**N**	**Death, n (%)**	**Time to death (months), median (95% CI)**	**KM estimate at 24 months, % (95% CI)**
**Overall**	183	97 (53.0)	29.9 (17.8–NE)	52 (44–59)	183	57 (31.1)	20.1 (11.9–58.2)	41 (30–53)
**LFR**	60	19 (31.7)	NE (NE–NE)	71 (58–81)	60	10 (16.7)	NE (14.6–NE)	57 (34–75)
**S1**	108	55 (50.9)	31.7 (17.8–NE)	53 (43–62)	108	33 (30.6)	14.6 (9.6–NE)	36 (22–50)
**S2+**	58	33 (56.9)	13.0 (8.6–NE)	48 (35–60)	58	19 (32.8)	20.4 (4.4–NE)	41 (19–63)
**Prior allo-HSCT**	38	16 (42.1)	74.7 (22.1–NE)	65 (48–78)	38	9 (23.7)	NE (12.3–NE)	72 (49–86)
**No prior allo-HSCT**	145	81 (55.9)	22.6 (12.7–NE)	49 (40–57)	145	48 (33.1)	12.2 (8.7–20.4)	28 (16–42)

allo-HSCT, allogeneic haematopoietic stem cell transplant; BCP-ALL, B-cell precursor acute lymphoblastic leukaemia; CI, confidence interval; KM, Kaplan–Meier; LFR, late first relapse; NE, not estimable; Ph, Philadelphia chromosome; R/R, relapsed/refractory; S1, patients who received blinatumomab as first salvage therapy; S2+, patients who received blinatumomab as subsequent salvage therapy.

Supplementary Table 9. HSCT following blinatumomab treatment in adult patients with MRD+ Ph− BCP-ALL

	**Adult MRD+ Ph− BCP-ALL (N=46)**
**Allo-HSCT status, n (%)**	
Received after blinatumomab therapy	33 (71.7)
Without anti-cancer therapy prior to allo-HSCT	28 (84.8)
With anti-cancer therapy prior to allo-HSCT	5 (15.2)
Did not receive	3 (6.5)
Not available	10 (21.7)
**Intensity of conditioning regimen, n (%)**	
Myeloablative	23 (69.7)
Reduced intensity/non-myeloablative	4 (12.1)
Not available	6 (18.2)
**Time to transplant, median in months (min–max)**	
Among patients (n=28) without anti-cancer therapy prior to allo-HSCT	2.4 (0.3–6.4)
Among patients (n=5) with anti-cancer therapy prior to allo-HSCT	6.2 (1.4–8.7)
**Mortality after allo-HSCT (overall)**	**N=33**
Death, n (%)	9 (27.3)
Time to death (months), median (min–max)	NE (1.4–32.3)
**Mortality after allo-HSCT in patients without anti-cancer therapy prior to allo-HSCT**	**N=28**
Death, n (%)	5 (17.9)
Relapse-related	4 (14.3)
Non–relapse-related	1 (3.6)
Time to death (months), median (min–max)	NE (1.6–32.3)

allo-HSCT, allogeneic haematopoietic stem cell transplant; BCP-ALL, B-cell precursor acute lymphoblastic leukaemia; max, maximum; min, minimum; NE, not estimable; Ph, Philadelphia chromosome; R/R, relapsed/refractory.

Supplementary Table 10. HSCT following blinatumomab treatment in adult patients with R/R Ph− B-ALL

	Adult R/R Ph− BCP-ALL (N=183)	Adult R/R Ph− BCP-ALL LFR (N=60)
**Allo-HSCT status after blinatumomab, n (%)**		
Received	101 (55.2)	39 (65.0)
As first allo-HSCT	83 (82.2)	29 (74.4)
In patients who achieved CR/CRh/CRi and received	76 (75.2)	25 (64.1)
First allo-HSCT without anti-cancer therapy	56	23
First allo-HSCT with anti-cancer therapy	20	2
After a prior allo-HSCT	18 (17.8)	10 (25.6)
In patients who achieved CR/CRh/CRi and received	16 (88.9)	8 (80)
Allo-HSCT without anti-cancer therapy	8	4
Allo-HSCT with anti-cancer therapy	8	4
Did not receive	31 (16.9)	10 (16.7)
Not available	51 (27.9)	11 (18.3)
**Intensity of conditioning regimen among patients receiving allo-HSCT, n (%)**	**N=101**	**N=39**
Myeloablative	66 (65.3)	25 (64.1)
Reduced intensity/non-myeloablative	16 (15.8)	4 (10.3)
Not available	21 (20.8)	10 (25.6)
**Allo-HSCT procedure in patients achieving CR/CRh/CRi, n (%)**		
Without anti-cancer therapy prior to allo-HSCT	64 (68.8)	27 (79.4)
With anti-cancer therapy prior to allo-HSCT	28 (30.1)	6 (17.6)
**Allo-HSCT procedure in patients without CR/CRh/CRi, n (%)**		
With anti-cancer therapy prior to allo-HSCT	8 (100%)	5 (100%)

	Adult R/R Ph− BCP-ALL (N=183)	Adult R/R Ph− BCP-ALL LFR (N=60)
**Time to transplant, median in months (min–max)**		
In CR/CRh/CRi and no anti-cancer therapy prior to allo-HSCT	2.4 (0.2–10.1)	2.6 (0.3–4.6)
In CR/CRh/CRi and anti-cancer therapy prior to allo-HSCT	8.4 (0.3–36.8)	NE (2.8–36.8)
CR/CRh/CRi not achieved and anti-cancer therapy prior to allo-HSCT	8.5 (2.5–8.5)	5.0 (2.5–7.3)
**Mortality after allo-HSCT overall**		
Death, n (%)	40 (39.6)	9 (23.1)
Time to death, median in months (min–max)	NE (0.5–72.0)	NE (0.5–37.9)
**Mortality after allo-HSCT in patients in CR/CRh/CRi without anti-cancer therapy prior to allo-HSCT**	**N=64**	**N=27**
Death, n (%)	22/64 (34.4)	6/27 (22.2)
In patients with prior allo-HSCT	3/8 (37.5)	1/4 (25.0)
Relapse-related	1/8 (12.5)	1/4 (25.0)
Non–relapse-related	2/8 (25.0)	0/4 (0.0)
In patients with no prior allo-HSCT	19/56 (33.9)	5/23 (21.7)
Relapse-related	8/56 (14.3)	3/23 (13.0)
Non–relapse-related	11/56 (19.6)	2/23 (8.7)
Time to death, median in months (min–max)	NE (0.5–37.9)	NE (0.5–37.9)
Among patients with prior allo-HSCT	NE (3.7–18.6)	NE (18.6–18.6)
Among patients with no prior allo-HSCT	NE (0.5–37.9)	NE (0.5–37.9)

allo-HSCT, allogeneic haematopoietic stem cell transplant; BCP-ALL, B-cell precursor acute lymphoblastic leukaemia; CR, complete remission with full recovery of peripheral blood counts; CRh, complete remission with partial recovery of peripheral blood counts; CRi, complete remission with incomplete recovery of peripheral blood counts; LFR, late first relapse; max, maximum; min, minimum; NE, not estimable; Ph, Philadelphia chromosome; R/R, relapsed/refractory.

Supplementary Table 11. Safety outcomes

AEs, n (%)	Full analysis set[a] (N=264)		Adult MRD+ Ph− BCP-ALL (N=46)		Adult R/R Ph− BCP-ALL (N=183)	
	TEAE	TR-TEAE	TEAE	TR-TEAE	TEAE	TR-TEAE
**Any grade**	240 (90.9)	182 (68.9)	39 (84.8)	32 (69.6)	170 (92.9)	127 (69.4)
**Grade ≥3**	153 (58.0)	91 (34.5)	18 (39.1)	12 (26.1)	111 (60.7)	64 (35.0)
**Grade ≥4**	71 (26.9)	31 (11.7)	7 (15.2)	4 (8.7)	55 (30.1)	24 (13.1)
**Serious**	139 (52.7)	77 (29.2)	17 (37.0)	12 (26.1)	98 (53.6)	50 (27.3)
**Fatal**	20 (7.6)	2 (0.8)	3 (6.5)	1 (2.2)	15 (8.2)	1 (0.5)
**Leading to drug interruption**	83 (31.4)	66 (25.0)	10 (21.7)	8 (17.4)	65 (35.5)	52 (28.4)
**Leading to drug discontinuation**	26 (9.8)	13 (4.9)	2 (4.3)	0 (0)	20 (10.9)	10 (5.5)
**AEs of interest, n (%)**						
**Neurologic AEs**	110 (41.7)	74 (28.0)	19 (41.3)	16 (34.8)	77 (42.1)	47 (25.7)
Grade ≥3	28 (10.6)	21 (8.0)	4 (8.7)	4 (8.7)	22 (12.0)	15 (8.2)
Grade ≥4	4 (1.5)	3 (1.1)	0 (0)	0 (0)	3 (1.6)	2 (1.1)
Serious	26 (9.8)	21 (8.0)	3 (6.5)	3 (6.5)	20 (10.9)	15 (8.2)
Fatal	1 (0.4)	1 (0.4)	0 (0)	0 (0)	1 (0.5)	1 (0.5)
Leading to drug interruption	31 (11.7)	30 (11.4)	6 (13.0)	6 (13.0)	22 (12.0)	21 (11.5)
Leading to drug discontinuation	8 (3.0)	7 (2.7)	0 (0)	0 (0)	6 (3.3)	5 (2.7)
**Cytokine release syndrome**	39 (14.8)	38 (14.4)	5 (10.9)	5 (10.9)	30 (16.4)	29 (15.8)
Grade ≥3	10 (3.8)	10 (3.8)	0 (0)	0 (0)	10 (5.5)	10 (5.5)
Grade ≥4	0 (0)	0 (0)	0 (0)	0 (0)	0 (0)	0 (0)
Serious	25 (9.5)	24 (9.1)	2 (4.3)	2 (4.3)	19 (10.4)	18 (9.8)
Fatal	0 (0)	0 (0)	0 (0)	0 (0)	0 (0)	0 (0)
Leading to drug interruption	14 (5.3)	14 (5.3)	0 (0)	0 (0)	14 (7.7)	14 (7.7)

AEs, n (%)	Full analysis set[a] (N=264)		Adult MRD+ Ph− BCP-ALL (N=46)		Adult R/R Ph− BCP-ALL (N=183)	
	TEAE	TR-TEAE	TEAE	TR-TEAE	TEAE	TR-TEAE
Leading to drug discontinuation	0 (0)	0 (0)	0 (0)	0 (0)	0 (0)	0 (0)
**Opportunistic infections**	9 (3.4)	4 (1.5)	2 (4.3)	1 (2.2)	6 (3.3)	3 (1.6)
Grade ≥3	4 (1.5)	1 (0.4)	1 (2.2)	0 (0)	3 (1.6)	1 (1.7)
Grade ≥4	1 (0.4)	0 (0)	0 (0)	0 (0)	1 (0.5)	0 (0)
Serious	7 (2.7)	3 (1.1)	2 (4.3)	1 (2.2)	4 (2.2)	2 (1.1)
Fatal	1 (0.4)	0 (0)	0 (0)	0 (0)	1 (0.5)	0 (0)
Leading to drug interruption	0 (0)	0 (0)	0 (0)	0 (0)	0 (0)	0 (0)
Leading to drug discontinuation	1 (0.4)	0 (0)	0 (0)	0 (0)	1 (0.5)	0 (0)

AE, adverse event; BCP-ALL, B-cell precursor acute lymphoblastic leukaemia; MRD, measurable residual disease; Ph, Philadelphia chromosome; R/R, relapsed/refractory; TEAE, treatment-emergent adverse event; TR-TEAE, treatment-related treatment-emergent adverse event.

[a]The full analysis set included an additional 35 patients with Ph+ BCP-ALL: nine with Ph+ R/R BCP-ALL (eight adult patients, one paediatric patient), five adult patients with Ph+ MRD+ BCP-ALL, and 21 with other ALL diagnoses.

Supplementary Table 12. Incidence of adverse events by age

	≥18 and <65 Years (N=227)	≥65 Years (N=36)
**Any-grade TEAEs, n (%)**	**203 (89.4)**	**36 (100.0)**
Grade ≥3	125 (55.1)	27 (75.0)
Grade ≥4	58 (25.6)	13 (36.1)
Serious	111 (48.9)	27 (75.0)
Fatal	14 (6.2)	6 (16.7)
Leading to drug interruption	64 (28.2)	19 (52.8)
Leading to drug discontinuation	21 (9.3)	5 (13.9)
**Any-grade TR-TEAEs, n (%)**	**150 (66.1)**	**31 (86.1)**
Grade ≥3	73 (32.2)	18 (50.0)
Grade ≥4	26 (11.5)	5 (13.9)
Serious	58 (25.6)	19 (52.8)
Fatal	1 (0.4)	1 (2.8)
Leading to drug interruption	51 (22.5)	15 (41.7)
Leading to drug discontinuation	10 (4.4)	3 (8.3)
**TEAEs of interest, n (%)**		
**Neurologic adverse events**	92 (40.5)	18 (50.0)
Grade ≥3	23 (10.1)	5 (13.9)
Grade ≥4	2 (0.9)	2 (5.6)
Serious	18 (7.9)	8 (22.2)
Fatal	0 (0.0)	1 (2.8)
Leading to drug interruption	22 (9.7)	9 (25.0)
Leading to drug discontinuation	6 (2.6)	2 (5.6)
**Cytokine release syndrome**	33 (14.5)	6 (16.7)
Grade ≥3	9 (4.0)	1 (2.8)

	≥18 and <65 Years (N=227)	≥65 Years (N=36)
Grade ≥4	0 (0.0)	0 (0.0)
Serious	20 (8.8)	5 (13.9)
Fatal	0 (0.0)	0 (0.0)
Leading to drug interruption	12 (5.3)	2 (5.6)
Leading to drug discontinuation	0 (0.0)	0 (0.0)
**Opportunistic infections**	9 (4.0)	0 (0.0)
Grade ≥3	4 (1.8)	0 (0.0)
Grade ≥4	1 (0.4)	0 (0.0)
Serious	7 (3.1)	0 (0.0)
Fatal	1 (0.4)	0 (0.0)
Leading to drug interruption	0 (0.0)	0 (0.0)
Leading to drug discontinuation	1 (0.4)	0 (0.0)

TEAE, treatment-emergent adverse event; TR-TEAE, treatment-related treatment-emergent adverse event.

Supplementary Table 13. Description of medication errors in the study

	Full Analysis Set[a] (N=264)	Adult MRD+ Ph− BCP-ALL (N=46)	Adult R/R Ph− BCP-ALL (N=183)	Adult R/R Ph− BCP-ALL LFR (N=60)
**≥1 medication error, n (%)**	26 (9.8)	6 (13.0)	15 (8.2)	7 (11.7)
1 medication error	22 (8.34)	4 (8.7)	13 (7.1)	6 (10.0)
2 medication errors	3 (1.1)	2 (4.3)	1 (0.5)	0 (0.0)
3 medication errors	1 (0.4)	0 (0)	1 (0.5)	1 (1.7)
**Total medication errors, n**	31	8	18	9
**Where did the error occur, n (%)**				
Inpatient	16 (51.6)	5 (62.5)	10 (55.6)	7 (77.8)
Outpatient clinic	6 (19.4)	3 (37.5)	2 (11.1)	1 (11.1)
Outside a medical facility	9 (29.0)	0 (0.0)	6 (33.3)	1 (11.1)
**Type of medication error, n (%)**				
Preparation	1 (3.2)	0 (0)	1 (5.6)	0 (0)
Administration	22 (71.0)	6 (75.0)	12 (66.7)	6 (66.7)
Product storage	0 (0)	0 (0)	0 (0)	0 (0)
Accidental exposure	0 (0)	0 (0)	0 (0)	0 (0)
Other	8 (25.8)	2 (25.0)	5 (27.8)	3 (33.3)
**If administration error, who was responsible for the error, n (%)**				
Operator use error	12 (38.7)	4 (50.0)	6 (33.3)	4 (44.4)
Pump malfunction	7 (22.6)	1 (12.5)	5 (27.8)	2 (22.2)
Line flush	0 (0)	0 (0)	0 (0)	0 (0)
Other	2 (6.5)	0 (0)	0 (0)	0 (0)
Unknown	1 (3.2)	0 (0)	1 (5.6)	0 (0)

BCP-ALL, B-cell precursor acute lymphoblastic leukaemia; LFR, late first relapse; MRD, measurable residual disease; Ph, Philadelphia chromosome; R/R, relapsed/refractory.

[a]The full analysis set included an additional 35 patients with Ph+ BCP-ALL: nine with Ph+ R/R BCP-ALL (eight adult patients, one paediatric patient), five adult patients with Ph+ MRD+ BCP-ALL, and 21 with other ALL diagnoses.
